# Baseline Platelet Activation and Reactivity in Patients with Critical Limb Ischemia

**DOI:** 10.1371/journal.pone.0131356

**Published:** 2015-07-06

**Authors:** Peter Paul Wisman, Martin Teraa, Gert Jan de Borst, Marianne C. Verhaar, Mark Roest, Frans L. Moll

**Affiliations:** 1 Department of Vascular Surgery, University Medical Center Utrecht, Utrecht, 3584CX, The Netherlands; 2 Department of Clinical Chemistry and Haematology, University Medical Center Utrecht, Utrecht, 3584CX, The Netherlands; 3 Department of Nephrology & Hypertension, University Medical Center Utrecht, Utrecht, 3584CX, The Netherlands; University of Edinburgh, UNITED KINGDOM

## Abstract

**Background:**

Patients with critical limb ischemia (CLI) have a high risk to develop cardiovascular events (CVE). We hypothesized that in CLI patients platelets would display increased baseline activation and reactivity.

**Objectives:**

We investigated baseline platelet activation and platelet reactivity in patients with CLI.

**Patients/Methods:**

In this study baseline platelet activation and platelet reactivity in response to stimulation of all major platelet activation pathways were determined in 20 CLI patients (11 using aspirin and 9 using vitamin K-antagonists) included in the Juventas-trial (clinicaltrials.gov NCT00371371) and in 17 healthy controls. Platelet activation was quantified with flow cytometric measurement of platelet P-selectin expression and fibrinogen binding.

**Results:**

CLI patients not using aspirin showed higher baseline platelet activation compared to healthy controls. Maximal reactivity to stimulation of the collagen and thrombin activation pathway was decreased in CLI patients compared to healthy controls. In line, attenuated platelet reactivity to stimulation of multiple activation pathways was associated with several traditional risk factors for cardiovascular disease.

**Conclusions:**

Baseline platelet activation was increased in CLI patients, whereas the reactivity of circulating platelets to several stimulatory agents is decreased. Reactivity of platelets was inversely correlated with cardiovascular risk factors.

## Introduction

Critical Limb Ischemia (CLI), the most advanced stage of peripheral artery disease (PAD), is characterized by ischemic rest pain or tissue loss as well as a profound risk for cardiovascular complications and mortality [1,2]. Abnormal platelet function with an increased tendency to aggregate is implicated in the pathogenesis of atherosclerosis [3] and development of superimposed acute ischemic events [4–7]. Antiplatelet therapy reduces the risk for future cardiovascular events (CVE) in patients with previous cardiovascular disease and is therefore the cornerstone of medical therapy in PAD [8]. Most commonly prescribed antiplatelet agents specifically inhibit platelet thromboxane production (aspirin) or platelet activation via the ADP-receptor (thienopyridines) and high-on-treatment platelet reactivity is associated with higher risk of future CVE [9]. To properly interpret platelet reactivity tests and employ effective interventions, more detailed data on platelet function in patients with severe PAD is mandatory. Conflicting results have been reported regarding platelet reactivity in PAD, possibly related to different patterns of platelet reactivity in different stages of PAD [10–12]. More research on platelet function is warranted to further elucidate the role of platelets in patients with severe PAD.

We hypothesized that CLI patients display increased baseline platelet activation and higher platelet reactivity than healthy controls, which may contribute to their increased cardiovascular risk. Platelet reactivity was assessed as P-selectin expression and fibrinogen binding, which reflects αIIBβ3 activation, using a flow cytometry based method. P-selectin and fibrinogen binding were measured in CLI patients at baseline (baseline platelet activation) and in response to stimulation of all major platelet activation pathways i.e. thrombin, collagen, ADP, and thromboxane activation pathway (platelet reactivity) [2].

To investigate platelet function in patients with severe PAD, we compared baseline platelet activation and platelet reactivity of patients with CLI with healthy controls.

## Methods

### Study subjects

20 patients with documented CLI, included in the Juventas-trial; a clinical trial evaluating the clinical effects of intra-arterial infusion of bone marrow mononuclear cells in CLI (clinicaltrials.gov NCT00371371), were included for the present study [13]. In short, the Juventas-trial included patients with chronic CLI, an ankle-brachial index (ABI) of 0.6 or less, or an unreliable index (non-compressible or not in proportion to the Fontaine classification), who were not candidate for conventional revascularization. Exclusion criteria were a history of neoplasm or malignancy in the past 10 years, concomitant disease with life expectancy of less than one year, inability to obtain sufficient bone marrow aspirate, known infection with human immunodeficiency virus, hepatitis B or C virus, and an impossibility to complete follow-up. In all 20 patients, 4.5 mL 3.2% tri-sodium citrate-anticoagulated venous blood samples were obtained before study interventions.

The antiplatelet therapy regimen was left to the discretion of the vascular surgeon and was recorded at inclusion and verified based on pharmaceutical supply records. For the remainder of the manuscript CLI patients not on antiplatelet therapy are referred to as CLI A- patients, patients using aspirin, as CLI A+ patients. Patients using clopidogrel or other platelet inhibitors were excluded for the present analysis.

Healthy controls, who did not use antiplatelet drugs for at least 7 days prior to blood withdrawal, were recruited from the mini donor service of the University Medical Center (UMC) Utrecht, consisting of healthy employees of the UMC Utrecht. Healthy controls were compared with the CLI A- patients. Ultimately, blood was obtained from 17 healthy controls, who were compared with 9 CLI A- patients. This study was conducted in accordance to the Declaration of Helsinki and procedures were approved by the institutional review board of the UMC Utrecht (Medisch Ethische Toetsingscommissie van het UMC Utrecht). All patients gave written informed consent.

### Study procedures

Platelet reactivity and baseline platelet activation was assessed within 90 minutes from blood withdrawal. Reactivity of platelets was determined with concentration series of: thrombin receptor agonist SFLLRN (TRAP) ranging from 0.038 to 625 μM, adenosine diphosphate (ADP) ranging from 0.008 to 125 μM, the thromboxane analog U-46619 ranging from 0.8 to 12500 μg/mL (Tx), convulxin (CVX) ranging from 0.13 to 80 μg/mL (modified procedure of previously published method [14]). Serial dilutions were prepared in 50 μL HEPES buffered saline (HBS; 10 mM HEPES, 150 mM NaCl, 1 mM MgSO4, 5 mM KCl, pH 7.4 4, 5 mM KCl, pH 7.4) with 2 μL phycoerythrin-labeled mouse α-human P-selectin antibodies and 1 μL fluorescein isothiocyanate-labeled mouse α-human fibrinogen antibodies. The platelet reactivity test was initiated by addition of 5 μL whole blood to each sample of the serial dilutions. After 20 min of incubation, the samples were fixed with 500 μL 0.2% formyl saline (0.2% formaldehyde in 0.9% NaCl) and kept at 4°C. All samples were analyzed on a FACS Calibur flow cytometer from BD Biosciences (Franklin Lakes, NJ, USA) within 1 day after processing. Single platelets were gated on the basis of forward-scatter and side-scatter properties, and their median fluorescence intensity (MFI) was measured. All assays were performed by a single observer blinded to subject characteristics.

### Study parameters

#### Platelet reactivity

For each individual subject, dose—response graphs and areas under the curves (AUC) expressed in arbitrary units were produced with PRISM software version 6.01 (Graphpad Software, La Jolla, CA, USA) for each agonist separately.

Baseline platelet activation was determined by averaging MFI’s from the lowest concentrations of the TRAP, ADP, CVX, and U-46619 concentration series.

### Statistics

Dichotomous variables are presented as frequencies and percentages and continuous variables are presented as means with standard deviation (SD). CLI A- patients were compared to healthy controls and to CLI A+ patients. Differences in baseline platelet activation between groups were tested with Student’s t-test. Differences in dose-response curves, maximal platelet reactivity and EC50 between groups were tested with extra-sum-of-squares F-test. Associations between patients’ baseline characteristics and platelet reactivity AUC’s were tested using the Spearman’s rank correlation tests. Statistical significant difference was considered at a two-sided *p*-value below 0.05. All analyses were performed using SPSS software version 20.0 (IBM, Chicago, IL, USA) and PRISM software version 6.01 (Graphpad Software, La Jolla, CA, USA).

## Results

### Baseline Characteristics

Twenty CLI patients and 17 healthy controls were included for this study. Mean age was 63.9±15.7 years for CLI patients and the majority of the CLI patients were Twenty male (70%; Table 1 and Database A in S1 file). The healthy controls were younger (45.1±9.6) and the majority was female (65%). The different groups of CLI patients were not different with respect to age, sex, cardiovascular history and medication use.

**Table 1:** Baseline Characteristics.

	CLI patients	Healthy controls
	(n = 20)	(n = 17)
Age (years)	63.9±15.7	45.1±9.6
Male gender	14 (70%)	6 (35%)
History of cardiovascular disease		
Previous revascularisation of affected leg	16 (80%)	-
CABG	4 (20%)	-
Myocardial infarction or angina pectoris	6 (30%)	-
TIA or stroke	4 (20%)	-
Cardiovascular risk factors		
Currently smoking	7 (35%)	-
Diabetes	8 (40%)	-
Hypertension	13 (65%)	-
Medication use		
Aspirin	11 (55%)	-
Anticoagulants	9 (45%)	-
Statins	17 (85%)	-
ACE inhibitors	7 (35%)	-
Angiotensin receptor blocker	2 (10%)	-
Beta-blockers	4 (20%)	-
Diuretics	5 (25%)	-
Laboratory parameters		
Total cholesterol (mmol/l)	4.2±1.2	-
HDL-cholesterol (mmol/l)	1.2±0.4	-
HbA1c (mmol/mol)	51.3±18.5	-
Homocysteine (μmol/l)	18.8±13.4	-
Creatinin (μmol/l)	104.6±39.2	-
Ureum (mmol/l)	7.6±4.4	-
Fontaine classification (grade IIB/III/IV)	1/3/16	-

Values are presented as absolute numbers and percentage (n [%]) for categorical variables and mean ± standard deviation (SD) for continuous variables. History of hypertension was defined as reported history of hypertension or being on antihypertensive medication.

### Baseline platelet activation is increased in CLI patients

**Fig 1:**
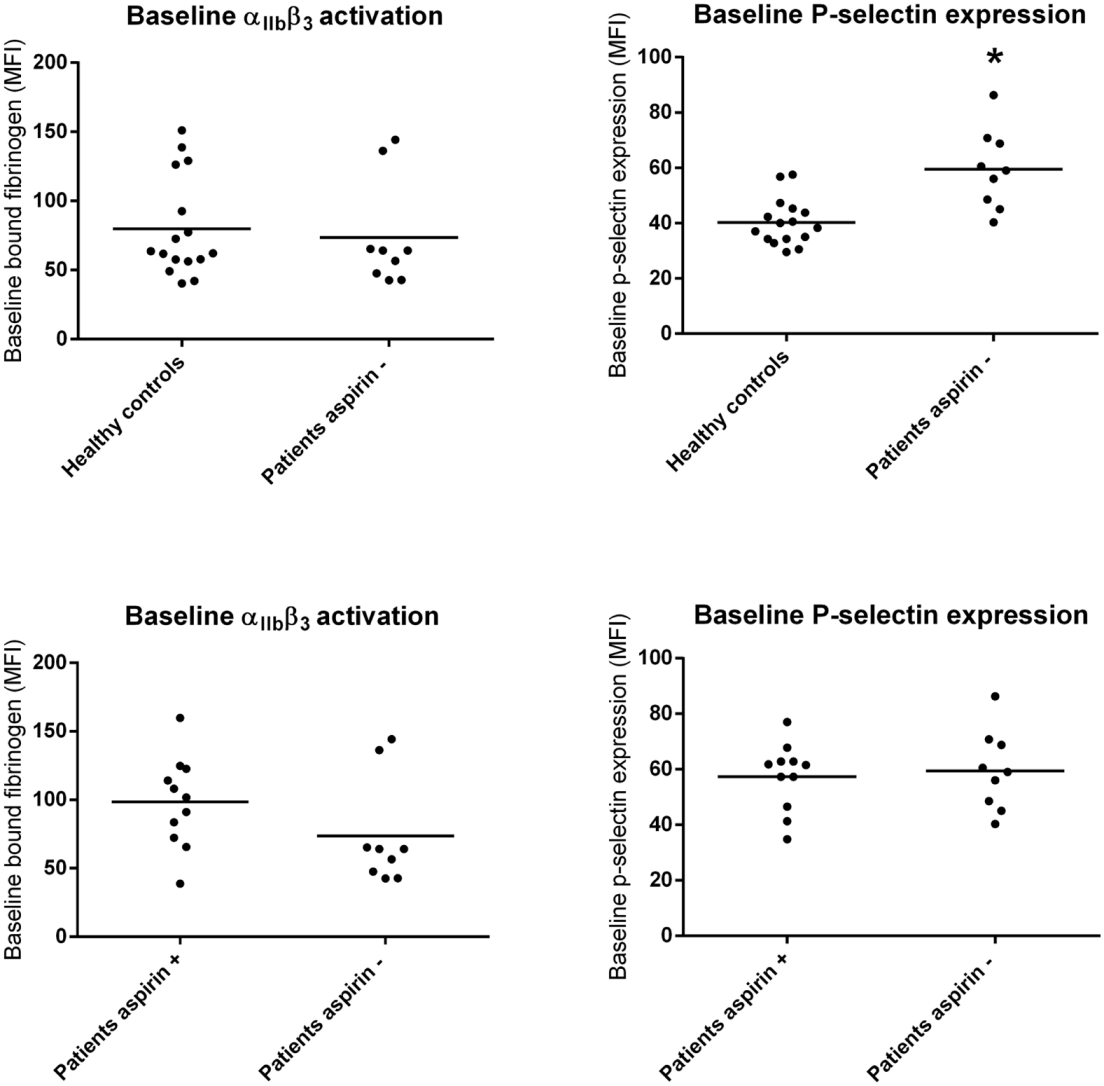
Baseline platelet activation of CLI A- patients versus healthy controls and CLI A- patients versus CLI A+ patients. MFI for bound fibrinogen or P-selectin expression without stimulation, stratified for CLI A- patients versus healthy controls and CLI A- patients versus CLI A+ patients. *MFI* median fluorescence intensity, * *p*<0.05.

Baseline platelet activation was determined with the P-selectin expression and the fibrinogen binding capacity of the patient-derived platelets without stimulation with an agonist. Baseline platelet α_IIB_β_3_ activation did not differ between CLI A- patients and healthy controls (73.7±12.9 vs. 80.3±9.7, *p* = 0.682). Baseline P-selectin expression was significantly higher in CLI patients compared to healthy controls (59.4±4.8 vs. 40.3±8.6, *p*<0.001; Fig 1 and Fig A in S1 file).

When CLI A- patients were compared to CLI A+ patients, no differences in baseline α_IIB_β_3_ activation (73.7±12.9 vs. 98.3±10.0, *p* = 0.142) and baseline P-selectin expression (59.4±4.8 vs. 57.3±12.2, *p* = 0.724) were observed (Fig 1).

### In-vitro platelet reactivity is reduced in CLI patients

**Fig 2:**
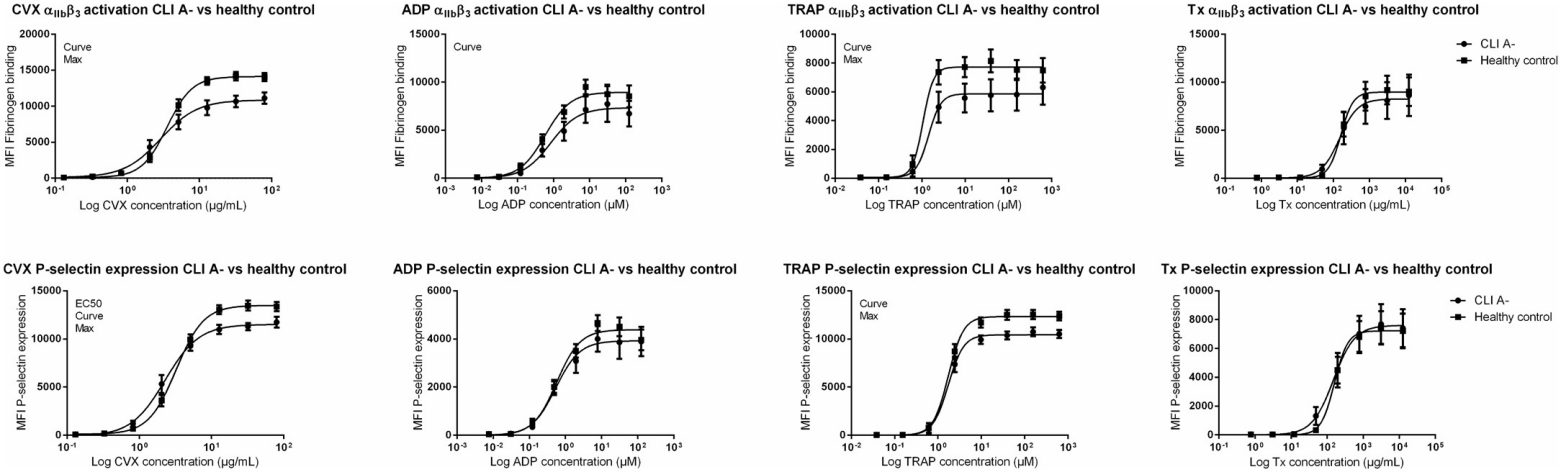
Platelet reactivity of CLI A- patients versus healthy controls. MFI for fibrinogen binding or P-selectin expression per agonist, stratified for CLI A- patients versus healthy controls. *CLI A-* CLI patients not treated with aspirin, *MFI* median fluorescence intensity, *CVX* Convulxin, *ADP* Adenosine Diphosphate, *TRAP* Thrombin receptor agonist SFLLRN, *Tx* Thromboxane receptor agonist, *Curve dose-response* curves of each group differ significantly *(p*<0.05), *Max* maximal platelet reactivity of each group differ significantly *(p*<0.05), *EC50* half maximal effective concentration of each group differ significantly *(p*<0.05).

Platelet reactivity was determined with P-selectin expression or the fibrinogen binding capacity after stimulation to four major platelet agonists. Overall, *in-vitro* platelet reactivity in CLI A- patients was not elevated for any of the agonists, when compared to healthy controls. Instead, CLI A- patients’ maximal platelet α_IIB_β_3_ activation and P-selectin expression was decreased for CVX (*p* = 0.001) and TRAP (*p* = 0,004) activation (Fig 2 and Fig B,C in S1 file). CLI A- patients had a lower EC50 platelet P-selectin expression for CVX activation than healthy donors (2.28±0.24 vs 3.25±0.21; *p* = 0.005).

**Fig 3:**
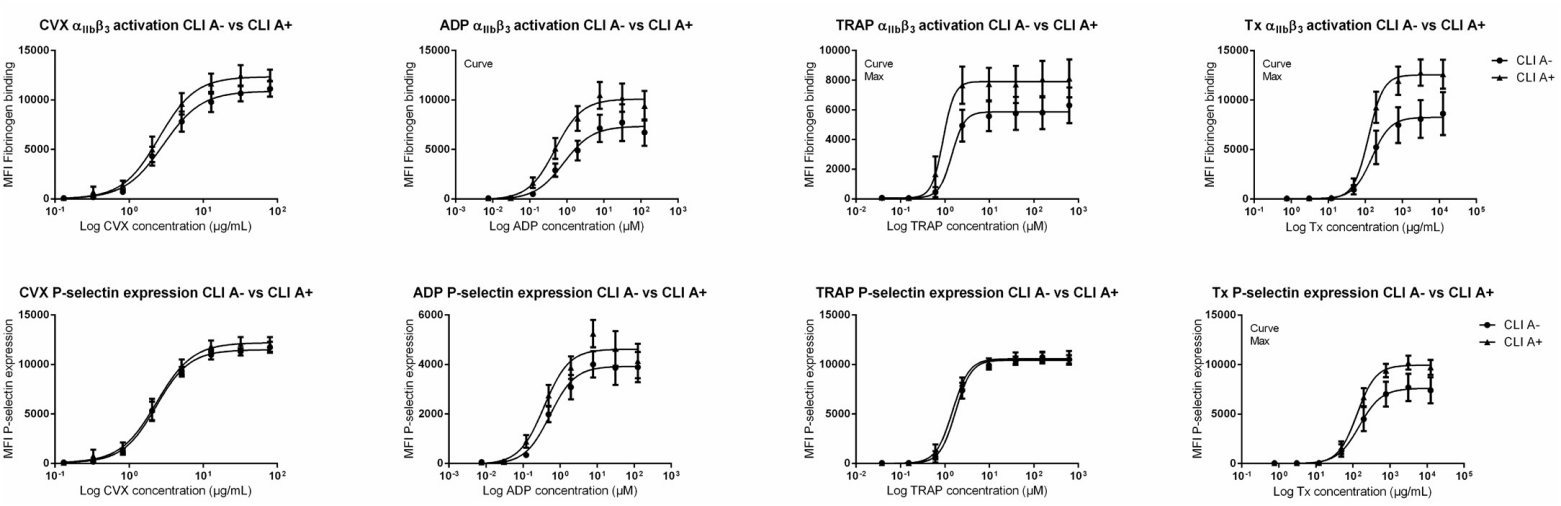
Platelet reactivity of CLI A- patients versus CLI A+ patients. MFI for fibrinogen binding or P-selectin expression per agonist, stratified for CLI A- patients versus CLI A+ patients. *CLI A-* CLI patients not treated with aspirin, *CLI A+* CLI patients treated with aspirin, *MFI* median fluorescence intensity, *CVX* Convulxin, *ADP* Adenosine Diphosphate, *TRAP* Thrombin receptor agonist SFLLRN, *Tx* Thromboxane receptor agonist, *Curve* curves for each group differ significantly *(p*<0.05), *Max* maximal platelet reactivity for each group differ significantly *(p*<0.05).

Platelet reactivity did differ between CLI A- patients and CLI A+ patients for the ADP, thrombin and thromboxane activation pathways (Fig 3 and Fig B,C in S1 file). Compared to CLI A+ patients, CLI A- patients’ maximal platelet α_IIB_β_3_ activation was decreased for Tx and TRAP activation. Maximal platelet P-selectin expression was decreased in CLI A- patients for Tx activation when compared to CLI A+ patients.

### Increased cardiovascular disease burden is associated with reduced in-vitro platelet reactivity

Associations between patient’s’ baseline characteristics and platelet reactivity parameters were tested in the CLI patients (CLI A- and CLI A+). Platelet reactivity was in general negatively correlated with a history of angina pectoris or myocardial infarction. An inverse trend was also observed for the correlation between platelet reactivity and markers for renal function, creatinine and urea. Age, presence of diabetes, HbA1c and homocysteine levels showed a tendency towards an inverse association with platelet response to several of the platelet activators (Table 2 and database A in S1 file).

**Table 2:** Correlations of patient factors with platelet reactivity.

			CVX		ADP		TRAP		Tx	
			Fibr	P-sel	Fibr	P-sel	Fibr	P-sel	Fibr	P-sel
Age			-0.24	-0.26	-0.29	-0.12	-0.26	-0.47*	-0.26	-0.43^†^
Sex			-0.08	0.17	-0.19	-0.10	0.38	0.19	0.08	0.21
Hypertension			-0.25	0.19	-0.25	0.16	-0.06	0.28	-0.28	0.28
Diabetes mellitus			-0.27	-0.27	-0.23	-0.21	-0.28	-0.41^†^	-0.35	-0.39^†^
HbA1c			-0.35	-0.07	-0.42	-0.26	-0.52^†^	-0.53^†^	-0.40	-0.29
Smoking			0.19	0.06	0.34	0.28	0.33	0.22	0.39^†^	0.31
History of AP or MI			-0.45*	-0.23	-0.42	0.02	-0.25	0.02	-0.28	0.17
History of CABG			-0.65**	-0.39^†^	-0.65**	-0.24	-0.48*	-0.15	-0.48*	-0.13
History of TIA or CVA			-0.35	-0.04	-0.41^†^	-0.15	-0.41^†^	-0.26	-0.28	-0.30
Previous revascularization			-0.13	0.11	-0.33	-0.15	-0.24	0.11	-0.22	-0.04
Fontaine classification			0.04	-0.11	0.10	0.24	0.22	0.10	0.34	0.43
Cholesterol			0.24	0.32	0.21	-0.09	0.08	0.09	0.03	-0.20
HDL			0.16	0.17	0.14	-0.10	0.22	0.35	0.23	0.23
Creatinin			-0.16	-0.28	-0.13	-0.18	-0.23	-0.66**	-0.31	-0.35
Ureum			-0.18	-0.31	-0.15	-0.17	-0.18	-0.60**	-0.20	-0.38^†^
Homocysteine			-0.38^†^	-0.31	-0.36	-0.34	-0.16	-0.01	-0.16	0.03
Number of platelets			-0.20	-0.07	-0.15	0.21	0.11	0.38	0.25	0.52*
Mean platelet volume			0.43^†^	0.15	0.44*	-0.07	0.15	-0.21	0.09	-0.25
Aspirin use			0.25	0.11	0.39	0.10	0.34	0.10	0.43^†^	0.36
Statin use			-0.16	-0.28	-0.16	-0.01	-0.09	-0.11	0.04	0.01
Use of Diuretics			-0.25	-0.13	-0.25	0.09	-0.15	0.09	-0.35	-0.03
Use of ACE-inhibitor			-0.46*	0.01	-0.63**	-0.12	-0.41^†^	-0.06	-0.46*	-0.17
Beta-blocker use			-0.28	-0.07	-0.28	0.13	-0.15	0.22	-0.17	0.17

Values represent Spearman’s rho or point-biserial correlation coefficients (r_pb_) in case one of the variables is categorical. *CVX* Convulxin, *ADP* Adenosine Diphosphate, *TRAP* Thrombin receptor agonist SFLLRN, *Tx* Thromboxane receptor agonist, *Fibr* Fibrinogen binding, *P-sel* P-selectin expression

^†^ *p*<0.10

* *p*<0.05

** *p*<0.01

## Discussion

Our study shows increased baseline activation of circulating platelets in CLI patients who are not on aspirin therapy. The reactivity of circulating platelets to simulation of the thrombin, ADP, and collagen activation pathway in CLI patients was different from healthy controls, maximal reactivity to stimulation of the collagen and thrombin activation pathway was attenuated in CLI patients compared to healthy controls. In line, attenuated platelet reactivity to stimulation of multiple activation pathways was associated with several traditional risk factors for cardiovascular disease.

Platelet adhesion to activated endothelial cells or the denuded vessel wall is an early event in the atherosclerotic process [15,16]. However, the exact role of platelets and platelet function in atherosclerotic progression is not established and their role in extensive atherosclerotic states, such as CLI, is still under debate [11,12,17,18]. Our findings of an increased baseline platelet P-selectin expression and a lack of difference in baseline platelet αIIBβ3 activation in patients with CLI compared to healthy controls are in line with the findings of Cassar et al. [11]. It is currently unknown what causes the observed divergence between baseline αIIBβ3 activation and baseline P-selectin expression. αIIBβ3 is involved in firm adhesion to activated endothelial cells, the subendothelial matrix, and platelet aggregation by fibrinogen and von Willebrand factor binding, while P-selectin is involved in initial rolling of platelets on the endothelial surface upon activation [19]. One possible explanation could be that platelets with moderate αIIBβ3 activation are captured from the circulation, while those with moderate P-selectin expression circulate for a prolonged period of time. Hence P-selectin expression of circulating platelets might provide a more realistic representation of the *in-vivo* platelet activity.

Our observations of a tendency to a decreased platelet reactivity to most agonists in patients with end-stage CLI compared to healthy controls seems in conflict with several other studies suggesting increased platelet reactivity in claudicants compared to healthy controls [11,17]. Interestingly, a similar decreased platelet reactivity to ADP pathway stimulation in CLI patients has been reported previously [11], in the current study we show that particularly maximal platelet reactivity is decreased in CLI patients. Additionally, the present study shows that *in-vitro* platelet reactivity is inversely associated with the burden of cardiovascular disease and risk, i.e. history of myocardial infarction or angina pectoris, decreased renal function, and elevated homocysteine and HbA1c levels, which is an additional indication for attenuated *in-vitro* reactivity of circulating platelets in patients with extensive atherosclerotic disease. Patients using aspirin showed *in-vitro* platelet reactivity more alike that of healthy controls, suggesting that aspirin might partly correct these changes in platelet reactivity.

The divergent results between claudicants and CLI patients suggest a different pattern of platelet reactivity in different PAD stages. It has been suggested that capture of the most reactive platelets by diseased endothelium and existing atherosclerotic plaques may result in a residual circulating pool of relatively activation resistant platelets [11]. Another potential explanation is that increased proteolytic shedding of surface receptors involved in platelet activation potentially down-regulates the platelet reactivity to its agonists [20–22], since proteases responsible for this surface receptor shedding are elevated in cardiovascular diseases [23,24]. Furthermore, subclinical intra-plaque hemorrhage is associated with progression of atherosclerotic lesions and may occur more frequently in patients with low platelet reactivity [2]. Subsequently, low platelet reactivity might contribute to progression of atherosclerotic lesions which could lead to CLI.

Our study has some limitations. First, our study was not randomized and therefore sensitive to confounding factors. We cannot exclude that differences in age and sex between CLI patients and healthy donors might have influenced our results. However, it is known that platelet reactivity in a healthy population is not dependent on age [25]. Platelet reactivity in females is in general higher [26], but no relation of age or sex with any of the platelet reactivity parameters was observed in our control population. There were no differences in baseline characteristics among the different subgroups of CLI patients. The cross-sectional design of our study does not allow conclusions on the relation of platelet reactivity to different agonists and future CVE risk; this should be investigated in future longitudinal studies. Additionally, our study has a relatively small sample size, low power and no pre-study power calculation could be performed. However, studying the platelet reactivity in such detail as in this study provides a basis for future focused studies in larger patient populations, which are mandatory to validate our result. Noteworthy, platelet reactivity was only assessed using flow cytometric analysis, which does not assess the speed of platelet activation. Addition of platelet aggregometry is likely to show similar results as both methods reflect α_IIB_β_3_ activation.

## Conclusion

Our study shows that CLI patients have increased baseline activation of circulating platelets compared to healthy controls, whereas the reactivity of circulating platelets to several stimulatory agents is decreased. Additionally, *in-vitro* reactivity of circulating platelets is inversely correlated to several established risk factors of cardiovascular disease. Prospective studies are required to investigate whether platelet reactivity to different agonists predicts future CVE in CLI patients and other populations at high risk for CVE.

## Supporting Information

S1 File Compressed zip file which consists of the study database and supporting data for Figs 1–3. Database A, Study database. Fig A, Supporting data for Fig 1. Fig B,C, Supporting data for Fig 2 and Fig 3.
(ZIP)
